# Ultrafast Formation of Small Polarons and the Optical Gap in CeO_2_

**DOI:** 10.1021/acs.jpclett.0c01590

**Published:** 2020-06-25

**Authors:** Jacopo Stefano Pelli Cresi, Lorenzo Di Mario, Daniele Catone, Faustino Martelli, Alessandra Paladini, Stefano Turchini, Sergio D’Addato, Paola Luches, Patrick O’Keeffe

**Affiliations:** † CNR-ISM, Division of Ultrafast Processes in Materials (FLASHit), Area della Ricerca di Roma 1, Istituto di Struttura della Materia-CNR (ISM-CNR), 00015 Monterotondo Scalo, Italy; ‡ Division of Ultrafast Processes in Materials (FLASHit), Area della Ricerca di Roma 2 Tor Vergata, Istituto di Struttura della Materia-CNR (ISM-CNR), Via del Fosso del Cavaliere 100, 00133 Rome, Italy; § Istituto per la Microelettronica e i Microsistemi-CNR, Area della Ricerca di Roma 2, IMM, Via del Fosso del Cavaliere 100, 00133 Rome, Italy; ∥ Dipartimento FIMUniversità degli Studi di Modena e Reggio Emilia, Via Campi 213/a, 41125 Modena, Italy; ⊥ CNR-NANO, Centro di Ricerca S3, via G. Campi 213/a, 41125 Modena, Italy

## Abstract

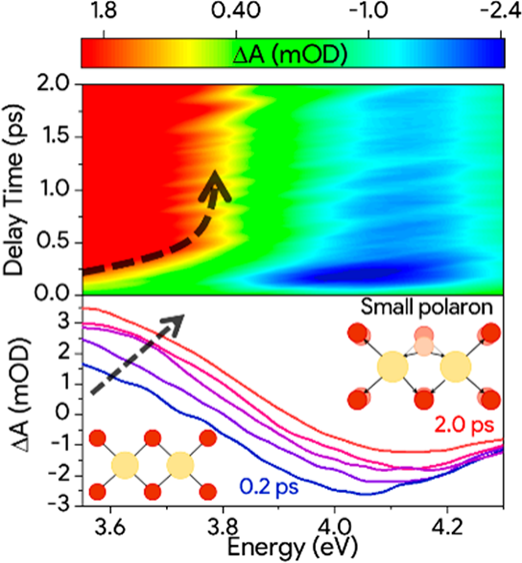

The ultrafast dynamics of excited states in cerium oxide are investigated to access the early moments of polaron formation, which can influence the photocatalytic functionality of the material. UV transient absorbance spectra of photoexcited CeO_2_ exhibit a bleaching of the band edge absorbance induced by the pump and a photoinduced absorbance feature assigned to Ce 4f → Ce 5d transitions. A blue shift of the spectral response of the photoinduced absorbance signal in the first picosecond after the pump excitation is attributed to the dynamical formation of small polarons with a characteristic time of 330 fs. A further important result of our work is that the combined use of steady-state and ultrafast transient absorption allows us to propose a revised value for the optical gap for ceria (*E*_og_ = 4 eV), significantly larger than usually reported.

Transition metal oxides (TMOs) are candidates for efficient photoelectrochemical catalysts of reactions such as water splitting and reduction of CO_2_.^1−4^ In such processes, an electron–hole pair is created by the absorption of a photon. The electrons can be used to promote reduction processes while the holes can be employed in oxidations, depending on whether the catalyst is used as photocathode or photoanode. The efficiency of these processes strongly depends on carrier lifetime and mobility.

CeO_2_, TiO_2_, and Fe_2_O_3_ are good catalysts because of their electronic structure, which allows transition metal ions to undergo redox cycles quickly and repeatably. However, the use of TMOs in photocatalysis is hampered by the formation of small polarons that affect the lifetime mobility of both charge carriers.^2,5−7^ A polaron is formed when the charge carriers polarize the lattice with the ensuing changes of the charge carrier energy.^8^ The polaron has a larger effective mass and smaller mobility than the bare charge carrier. Polarons are classified into two types depending on the spatial extension of the polarization field: large polarons are spread across several lattice unit cells while small polarons have the size of a single or few unit cells. The formation of small polarons has been observed in several TMOs such as Fe_2_O_3_,^9^ TiO_2_,^10^ NiO,^11^ and Co_3_O_4_.^12^ The textbook case of the small polaron model was proposed to explain electron mobility in partially reduced cerium oxide (CeO_2–*x*_).^5^ In particular, the presence of small polarons in CeO_2_ was suggested by the temperature dependence of the conductivity of single crystals and supported using the thermopower-conductivity relation.^13^ The results were interpreted as being due to an enhanced polarization field following the removal of oxygen atoms from the lattice.^14−16^ In this picture, the functionality of cerium oxide is linked to the mobility of oxygen ions, in turn entangled with carrier mobility via polaron hopping.^15,17^

While the transport mechanism of the ground-state polaron is established,^6^ only recently has the formation of small polarons in photoexcited states been studied,^2,18−20^ in particular in hemeatite^2,19^ and NiO.^11^ Those works have shown that the small polaron is formed by coupling between photoexcited electrons and the longitudinal optical (LO) phonons. For Fe_2_O_3_, a two-step process was proposed^2^ that takes place after photoexcitation by transferring electron density from oxygen to iron atoms. This initial coupling between excited electrons and optical phonons is followed by the recombination of the phonons with the hot electrons to form small polarons. The time constant for the process was found to be 660 fs in Fe_2_O_3_^19^ and 0.3–1.7 ps in NiO.^11^

Here, we suggest a similar process to occur in CeO_2_ based on time-resolved optical absorption measurements. We have taken advantage of the peculiar electronic structure of CeO_2_, which involves a valence band composed mainly of oxygen 2p states and a conduction band characterized by the cerium 5d states (Figure 1a).^21,22^ As shown in Figure 1a, localized unoccupied cerium 4f states lay between these bands.^21,23,24^ The UV light absorption of CeO_2_ induces a transition from the oxygen 2p states to the cerium 4f orbitals, which is allowed by the small but non-negligible hybridization between cerium and oxygen states.^21,25^ Here, using fast transient absorbance spectroscopy (FTAS), we explore the dynamics of the 4f states after the photoexcitation of CeO_2_. We observed a fast blue shift of the photoinduced transition from Ce 4f to the empty Ce 5d band. This behavior is interpreted as a modification of the band structure induced by small polaron formation (Figure 1b). Moreover, we will revisit the energy value of this 2p–4f transition as one of the outcomes of the comparison between steady-state and transient absorption measurements.

**Figure 1:**
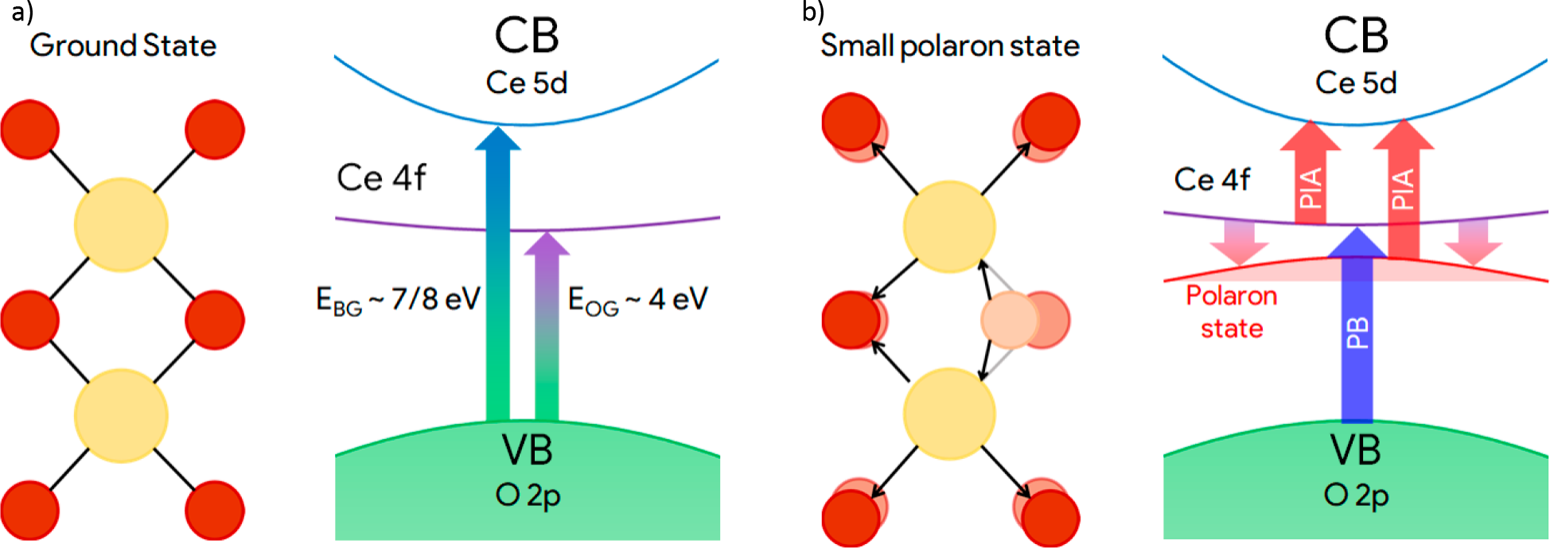
(a) Ground state of CeO_2_ characterized by a fluorite structure where Ce^4+^ (yellow) are near 8 O atoms (here simplified to 2 dimensions). Stoichiometric CeO_2_ presents a VB dominated by oxygen 2p states, a CB with a 7–8 eV bandgap and empty 4f states between these bands.^17,38^ The optical gap between Ce 4f states and VB is about 4 eV. (b) Photoexcitation of CeO_2_ inducing the filling of the Ce 4f states. This produces a polarization that deforms the lattice, causing the modification of the band structure. The energy state generated by the formation of small polaron state is schematized in red. The photobleachig and photoinduced absorption transient signal are highlighted with blue and red arrows.

We used a 6 nm thick film of CeO_2_ grown by molecular beam epitaxy (MBE) on quartz (SiO_2_) at room temperature by evaporating metallic cerium in a partial pressure of oxygen (10^–6^ mbar).^26^ The film was characterized using UV–vis spectrophotometry. The absorbance *A* was estimated by measuring the fraction of transmitted light *T* and of specular reflected light *R* (*A* = 1 – *T* – *R*), neglecting the scattered light. As shown in Figure 2, the quartz contribution to the absorbance is negligible up to 4.5 eV, while CeO_2_ exhibits a strong absorbance at energies higher than 3.2 eV with a shoulder at about 4 eV (highlighted by the dashed line). The analysis of Ce 3d XPS spectra taken *in situ* after the MBE deposition reports a superficial concentration of Ce^3+^ lower than 5%, showing the good stoichiometry of the film (see the Supporting Information).

**Figure 2:**
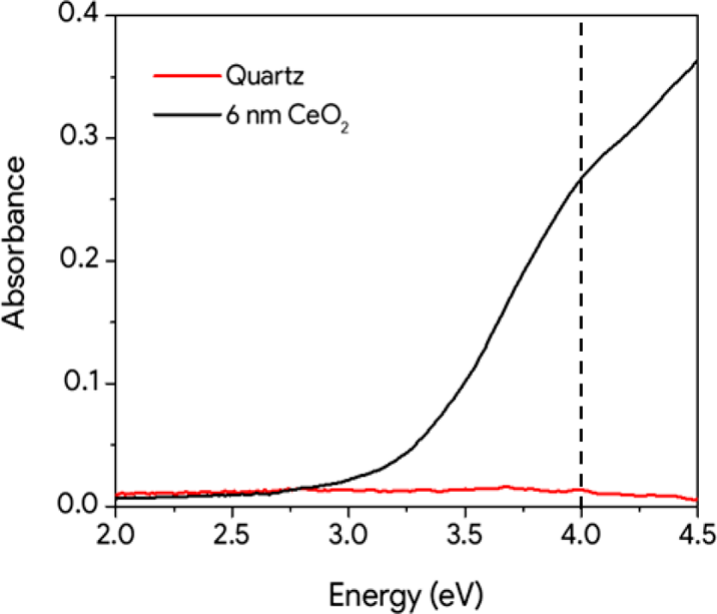
Absorbance of the CeO_2_ film (black) and of the quartz substrate (red). The dashed line highlights the shoulder of the absorbance ascribed to the optical gap of the material.

FTAS was used to probe the dynamics of the ceria optical response after an excitation induced by a pump pulse with energy above its optical bandgap. As a function of the pump–probe delay time, we measured the differences in absorbance of the sample when excited by the pump and when unperturbed, i.e. the transient absorbance Δ*A*. We probed the system using a visible (2.0–3.5 eV) or a UV (3.5–4.3 eV) supercontinuum. The instrument response function (IRF) has been evaluated in separate experiments to be characterized by a Gaussian with a FWHM of 70 fs^26−28^ (see the Experimental Section). Figure 3a shows the false-color maps of transient absorbance as a function of probe energy and delay-time after the photoexcitation. The two false-colored maps (VIS and UV) were joined at 3.55 eV as reported in Figure 3a. Transient spectra recorded at selected delay times (Figure 3b) show two main dominant signals in the UV region: a prevalent photobleaching (PB) centered at about 4 eV and a prevalent photoinduced absorption (PIA) centered at 3.55 eV.

**Figure 3:**
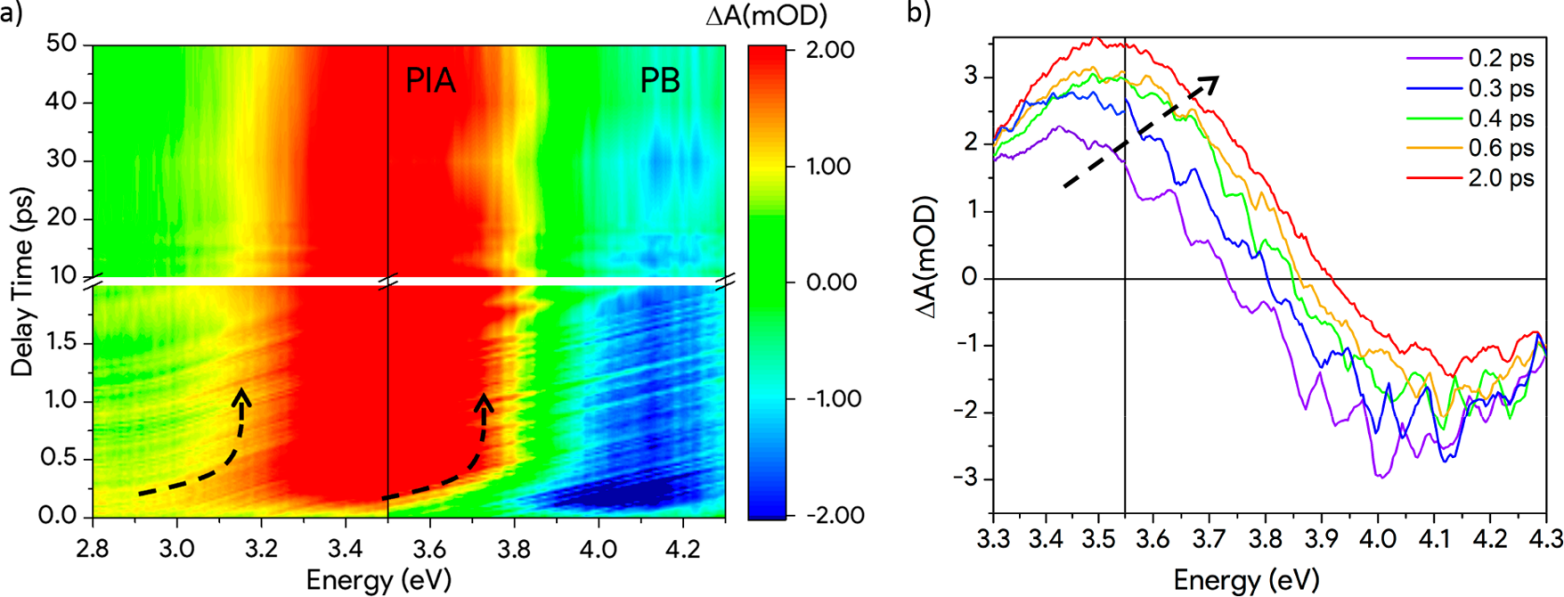
(a) False color transient absorbance map relative to the photoexcitation of the 6 nm thick CeO_2_ film. The low-energy part of the map was obtained using the visible probe setup (2.8–3.5 eV) while the high-energy part (3.5–4.3 eV) was obtained using the UV supercontinuum. The black arrows highlight the shift of the PIA band during the first picosecond. (b) Transient absorbance spectra of CeO_2_ in the UV region. The line at 3.55 eV represents the energy where the map recorded with the visible supercontinuum has been joined with the map recorded with the UV supercontinuum.

Before discussing the dynamics of Δ*A*, we bring the reader’s attention to the energy position of the PB peak. Usually, a PB signal indicates a bleaching of absorption induced by the pump and so a strong depletion of a ground state. In the first 200 fs, it occurs at about 4 eV (Figure 3b), very close to the energy of the shoulder observed in the steady-state absorbance shown in Figure 2. The independent observation of two absorption structures around the same energy leads us to suggest that the optical gap between O 2p and Ce 4f states of ceria is about 4 eV (schemes in Figure 1). This result is in contrast with almost all values reported in the literature. The optical gap of ceria, usually extracted via the Tauc method, lies in the range 3.0–3.6 eV.^22,25^ If we apply Tauc method to our steady-state spectrum, we obtain a gap of 3.55 eV (analysis in the Supporting Information). This value agrees with the literature, confirming that our absorption spectrum is typical of high-quality ceria, but is not consistent with the narrow and peaked PB signal in our FTAS spectra. The long absorption tail observed below 4.0 eV in the steady-state absorption should be considered as the Urbach tail, a very common feature of the absorption in defected semiconductors. It must be noted that defects in ceria induce the occupation of 4f localized states between the valence band and the 4f empty band, drastically modifying the absorption in the region of the Urbach tail. For these reasons, we propose 4 eV as the optical gap of ceria.

On the other side, on the basis of the CeO_2_ band structure (see Figure 1a), we assign the PIA signal between 3.2 and 3.7 eV (Figure 3b) to the transition from the photoexcited (by the pump) partially filled Ce 4f states to the unoccupied Ce 5d states (Figure 1b).^27^ The two signals seem to be involved in a rapid spectral change in the first picosecond (black dashed arrows in Figures 3), while at longer delay times they show only a simple decay behavior and a constant spectral shape.

The overlap between the positive PIA and negative PB signals in the UV region complicates the analysis and the deconvolution of the signals. Nevertheless, some qualitative explanations can be advanced. The visible portion of the PIA (2.8–3.5 eV), which is far from the PB signal, suggests that the PIA slightly shifts to higher energies in the first picosecond (as underlined by the black dashed arrows in Figure 3). The origin of this blue shift can be related to a lowering of the energy of photoexcited 4f electrons, and to a consequent increase of the energy required to further excite them into the conduction band. The decrease of the energy of the photoexcited 4f electrons is consistent with the formation of a small-polaron state, in analogy with the experimental observations on nonstoichiometric or donor-modified ceria and with theoretical predictions.^5,15,29,30^ Moreover, calculations by Sun et al.^17^ have demonstrated that localization on Ce is favorable over the delocalization of the electron across a large number of Ce 4f orbitals. For this reason, an excess electron in a supercell of bulk CeO_2_ relaxes principally to a nearly localized cerium state generating a local lattice polarization and so a small polaron. Photoinduced formation of small polarons has been reported in vis–XUV pump–probe experiments on similar oxides such as TiO_2_^10^ and α-Fe_2_O_3_.^2,9,18^ As in these cases, the high electron density transferred by the pump from O-like to Ce-like states could accelerate the interaction of the electrons with the lattice, thus forming the small polaron state.^2^

To extract quantitative information on the kinetics of the photoexcited ceria, we implemented a global analysis of the data using the Glotaran software.^31^ This approach is commonly used to extract the transient features of photoinduced components and their dynamics from the data helping to disentangle different contributions in FTAS measurements. To achieve a satisfactory fit of our data, it was sufficient to assume a sequential model in which an initially photoexcited state decays into a long-lived final state. The free parameters of the analysis are the shape of the spectral responses of two photoinduced components and their decay constants. The results of the global analysis are the two spectral components reported in Figure 4a (the black and red solid lines) characterized by the sequential exponential dynamics presented in Figure 4b. These must not be confused with the transient absorbance spectra (Figure 3b): the superposition of spectral components gives the best fit of the data. The goodness of the analysis is demonstrated by the comparison between the experimental and the Glotaran extracted temporal evolutions of different energies selected from the transient absorption measurements (Figure 4c). Furthermore, the residual FTAS map obtained by subtracting the model from the experimental data shows no evident features (see Figure S3c the Supporting Information).

**Figure 4:**
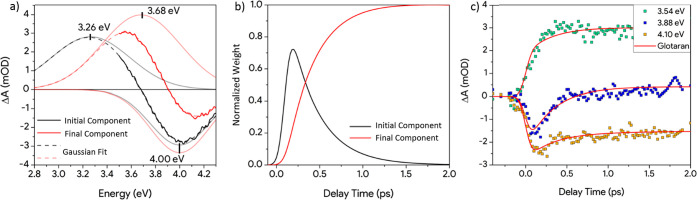
(a) Two spectral components extracted from the global analysis (solid lines). Each spectral component is fitted using two Gaussians (gray and light-red lines) in order to deconvolute the PIA and the PB contributions. The fits are reported with dashed lines. The centroid of the PIA and the PB are highlighted in the graph. (b) Weight dynamics of the two spectral components extracted by the global analysis. The sum of the weights is normalized to 1 outside the first few hundred femtoseconds where the IRF has a strong effect. (c) Comparison between the experimental dynamics at selected probe energies and the linear combination of the spectral components dynamics extracted with the Glotaran global analysis (red lines).

The black component in Figure 4a (identified as initial component) represents the first response of the system to the photoexcitation while the red one (identified as final component) defines the response at longer delay times (>2 ps). Both components are characterized by the same bleaching of the band edge together with the photoinduced absorption of the photoexcited system before and after the small polaron formation (as presented in Figure 1b). As the PB signal is related to VB depletion, it is expected to have constant energy as long as the excitation persists. Therefore, we fit each spectral component extracted with Glotaran with a sum of a positive (PIA) and a negative (PB) Gaussian. The resultant fit is reported with dashed lines in Figure 4a. Further details on the fitting procedure are reported in the Supporting Information. The centroid of the PIA-related Gaussian presents a shift of 0.42 eV (from 3.26 to 3.68 eV) that takes place in the first 2 ps. Following the literature, we assign this shift to the formation of the small polaronic state via the coupling between free electrons and the LO phonons of the lattice leading to states 0.4 eV below the unperturbed Ce 4f band.^16,17^ A similar behavior would have been observed also if the PIA energy shift would be due to an exciton formation instead of a polaron state. However, the energy formation that we measure, 0.4 eV, is compatible with the formation energy for a polaron calculated in literature^16,17^ and it appears too large to be an exciton binding energy in a polycrystalline material like ours. Such large binding energies are indeed observed only in 2D materials.^32^

The global analysis shows that the initial component (black curve in Figure 4b) rises in less than 70 fs (IRF of our system) and then decays with a time constant of 330 fs. The decay of this component results in the formation of the final component (red curve in Figure 4b), which shows a rise time of 330 fs and a decay time of 310 ps. Further details are reported in the Supporting Information. The dynamics of the first spectral component is compatible with the quick electron transfer from oxygen to metal states (O 2p → Ce 4f) which decays, compatibly with the dynamics of the first electron-optical phonon scattering/coupling events, into a small polaron state.^2,19,20^ These kinetics and the spectral blue shift confirm that the data are perfectly consistent with small polaron formation after photoexcitation.

The formation of small polarons after photoexcitation in CeO_2_ may have important consequences on its photoconductive and photochemical properties. It has been shown indeed that the presence of polarons affects oxygen vacancy formation and mobility,^33^ as well as the interaction with adsorbates.^34^ Understanding the dynamics of trapping and recombination of photogenerated electron–hole pairs is relevant and challenging. As these processes compete with charge transfer to adsorbed molecules and/or supported nanoparticles and with a transient alteration of the bonding strength between cerium and oxygen that influence reactivity and reducibility, as shown for similar oxides.^35,36^ Our study opens the way to more extensive investigations of photoexcited in CeO_2_-based materials aiming at understanding and optimizing the photoinduced functionalities.

To conclude, we have measured the steady-state and transient UV/vis absorbance of MBE-grown CeO_2_ thin film on quartz. The photoinduced transient UV/vis absorbance spectra revealed two features: a negative signal related to the bleaching of the band edge absorption and a positive signal we assigned to the re-excitation of the photoexcited Ce 4f electrons to the Ce 5d band. The analysis of the transient spectra allowed us to disentangle the dynamics of the formation of a small polaronic state and to determine its formation energy and time, being 0.4 eV and 330 fs, respectively. Moreover, we suggest the revised value of 4 eV for the optical band gap of ceria as the result of the combined use of steady-state and transient absorption spectra.

## Experimental Section

The cerium oxide film examined in this work was grown by molecular beam epitaxy (MBE) on a quartz (SiO_2_) substrate at room temperature by evaporating metallic cerium in a partial pressure of oxygen (10^–6^ mbar). This procedure, already described in previous works,^26^ was used to grow a 6 nm film of CeO_2_ with almost full stoichiometry. The film thickness was determined by using a cerium evaporation rate measured by a quartz crystal microbalance. The film stoichiometry was evaluated by in situ X-ray photoelectron spectroscopy (XPS) by fitting Ce 3d spectra using the procedure proposed by Skàla et al.^37^

Steady-state UV–vis spectrophotometry measurements were performed using a white nonpolarized light source generated by a xenon lamp equipped with an ORIEL-MS257 monochromator and a silicon photodetector (with a 250–750 nm range of detection). We estimated the absorbance *A* by measuring the fraction of transmitted light *T* and of specular reflected light *R* (*A* = 1 – *T* – *R*), neglecting the scattered light.

Our setup for the transient absorption spectroscopy is composed of a femtosecond laser system consisting of a chirped-pulse amplifier (800 nm, 1 kHz, 4 mJ, 35 fs) seeded by a Ti:Sa oscillator. As a pump, we used a 275 nm (4.5 eV) pulse generated by an optical parametric amplifier seeded by the amplifier. The fluence of the pump pulse was estimated to be 11 μJ/cm^2^. In order to generate the white light supercontinuum that acts as the probe in the visible range (2.00–3.60 eV), a small portion of the amplified fundamental 800 nm radiation (∼3 μJ) was focused into a rotating CaF_2_ crystal.^28^ The second harmonic of the amplified fundamental (400 nm) was used to drive the supercontinuum probe generation in the UV energy range (3.50–4.35 eV). In the transient absorbance maps presented in this work, the chirp of the probe pulse has been corrected. The instrument response function (IRF) has been evaluated in separate experiments to be Gaussian with a FHWM of 70 fs. Further details on the experimental setups are given elsewhere.^26,27^

## Supporting Information Available

Experimental section, analysis of Ce 3d XPS spectrum, Tauc analysis of optical absorption, global analysis of the FTAS map with residuals, and an analysis of the spectra extracted with the global fit analysis (PDF)
