# Supplementary material for: Ultrafast Formation of Small Polarons and the Optical Gap in CeO2
Source: J Phys Chem Lett. 2020 Jun 25;11(14):5686–91. doi: 10.1021/acs.jpclett.0c01590 (PMC8008440; doi:10.1021/acs.jpclett.0c01590)
Supplement: Supplementary file 1 — jz0c01590_si_001.pdf [file jz0c01590_si_001.pdf]

Supporting Information

Ultrafast Formation of Small Polarons and the Optical Gap in CeO_2

Jacopo Stefano Pelli Cresi [1,], Lorenzo Di Mario [2], Daniele Catone [2], Faustino Martelli [3], Alessandra Paladini [1], Stefano Turchini [2], Sergio D'Addato [4], Paola Luches [5], and Patrick O'Keeffe[1].*

1 Istituto di Struttura della Materia-CNR (ISM-CNR), Division of Ultrafast Processes in Materials (FLASHit), Area della Ricerca di Roma 1, 00015 Monterotondo Scalo, Italy.

2 Istituto di Struttura della Materia-CNR (ISM-CNR), Division of Ultrafast Processes in Materials (FLASHit), Area della Ricerca di Roma 2 Tor Vergata, Via del Fosso del Cavaliere 100, 00133 Rome, Italy.

3 IMM, Area della Ricerca di Roma 2 Tor Vergata, Via del Fosso del Cavaliere 100, 00133 Rome, Italy.

4 Università di Modena e Reggio Emilia, Via Campi 213/a, Modena, Italy.

5CNR-NANO, Centro di Ricerca S3, via G. Campi 213/a, Modena, Italy.

*Corresponding author

E-mail: jacopostefano.pellicresi@elettra.eu

Stoichiometry

The ceria surface stoichiometry for the CeO_2 film sample was evaluated by fitting the Ce 3d XPS spectra using Ce^{3+} and Ce^{4+} related components, following the procedure of Skala et al. [1]. The Ce 3d spectra of the two samples and the fitting curves are shown in Figure S1. The concentration of Ce^{3+} estimated in the sample is less than 5%, confirming a good stoichiometry close to CeO_2.

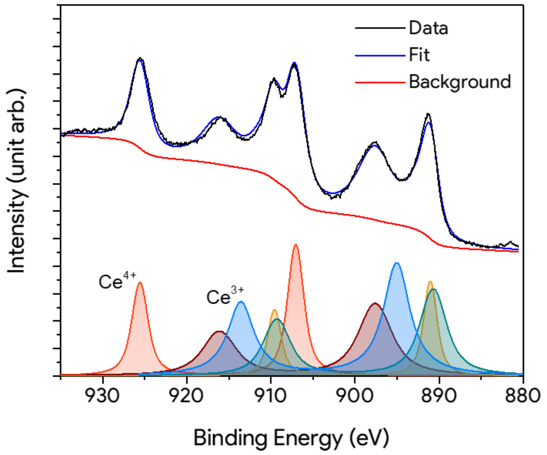

Figure S1 Ce 3d XPS spectrum. Black line is the experimental data while the blue line is the result of the fitting procedure. The fit was performed using a Shirley function for the background (in red) and the characteristic components for Ce3+ (highlighted by cold-colors) and Ce4+ (highlighted by warm-colors).

Optical Properties

We applied the Tauc's plot method to compare indirect and direct bandgap values obtained by fitting our experimental data with those found in the literature. This method assumes a linear behavior of the (α(hν)● hν)n vs. absorbed light energy plot as shown in Figure S2 to determine the direct (n=2) and indirect (n=1/2) band

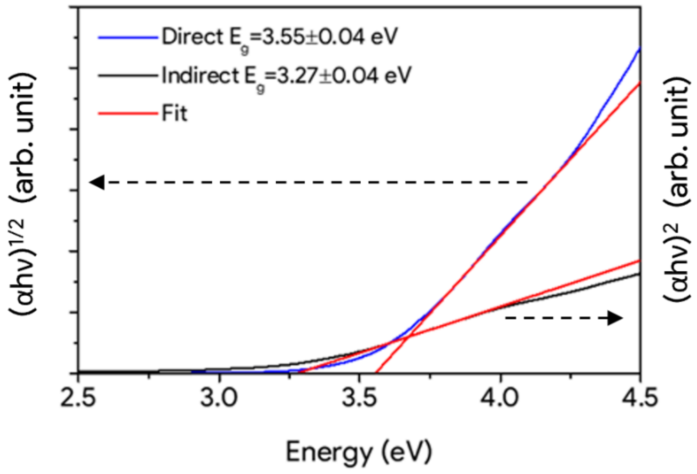

Figure S2 Comparison between the Tauc plot for the indirect optical band gap (in black) and for the direct one (in blue). In red we reported the linear fits used for the extraction of the indirect and direct optical band gaps.

gaps of the material from the optical absorption spectrum. While it is noted that the absorption in the sub-band gap region may be strongly affected by the defect density (as in the case of ZnO[2]) thus rendering extraction of the true direct and indirect band gaps questionable using this method, here we use the method purely as an indication of the quality of the CeO_2 sample by comparison to literature values extracted using the same method. In this way we determined a direct band gap of 3.55 ± 0.04 eV and an indirect band gap of 3.27 ± 0.04 eV. Both the results are comparable to those found in the literature [3,4]. However, as explained in the main text, we believe that the true bandgap corresponds to the shoulder of the absorbance at about 4 eV (as confirmed by the photobleaching at 4.0 eV in the fast transient measures).

Global Analysis

The global analysis provides a functional description of the kinetic profiles through exponential functions whose amplitudes are wavelength dependent. We performed this analysis using the Glotaran software[5]

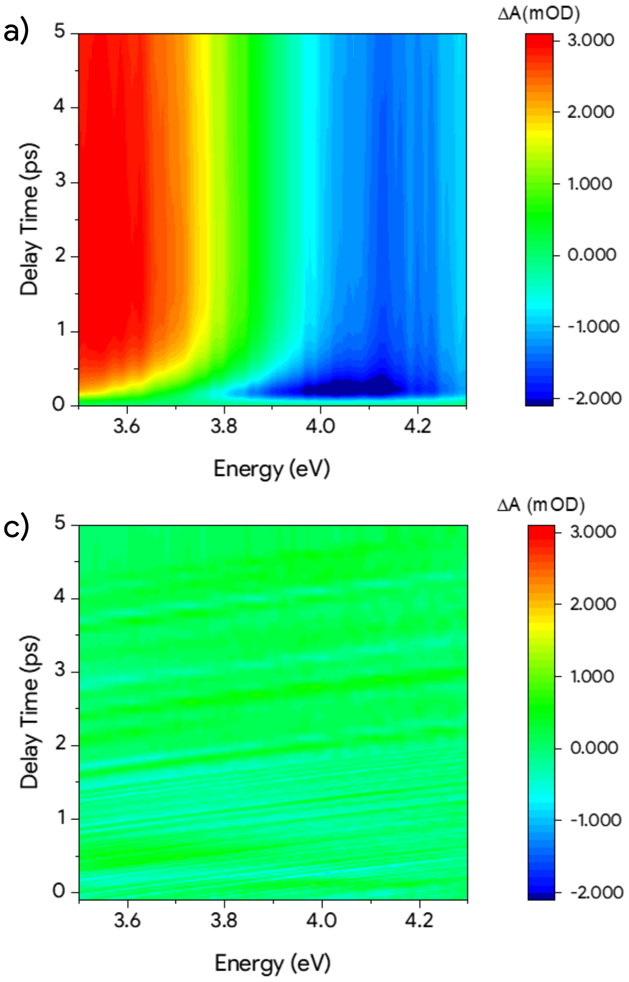

Figure S3 a. False color transient absorbance map obtained with the model extracted using global fit analysis. **b.** Experimental false color transient absorbance map. **c.** Residual map obtained by the subtraction of experimental map from the model map.

imposing two kinetic components with subsequent dynamics. An IRF with a FWHM of 70 fs (compatible with our system) was used in the analysis. The TA map obtained by this method (Figure S3a) is in good agreement with the experimental map (Figure S3b) as can be seen from residual map (Figure S3c).

Analysis of the spectra extracted with the Global Fit analysis

Two Gaussian peaks were used to simulate the PB (negative amplitude) and the PIA (positive amplitude) contributions to the spectral components extracted using the global analysis. As mentioned in the main text, the photobleaching Gaussian peak was fixed at 4 eV. Then the widths of the Gaussian peaks were fixed to the

Table S1 A1, B1, C1 are the intensity, the centroid and the width of the Gaussian for the PB signal while A2, B2, C2 the intensity, the centroid and the width of the PIA.

Initial Component				Final Component			
PB		PIA		PB		PIA	
A1	-2.94 mOD	A2	2.81 mOD	A1	-3.43 mOD	A2	3.97 mOD
B1	4 eV (fixed)	B2	3.26(2) eV	B1	4 eV (fixed)	B2	3.68 (2) eV
C1	0.35 (fixed)	C2	0.46 eV (fixed)	C1	0.35 (1) eV	C2	0.46 (1) eV

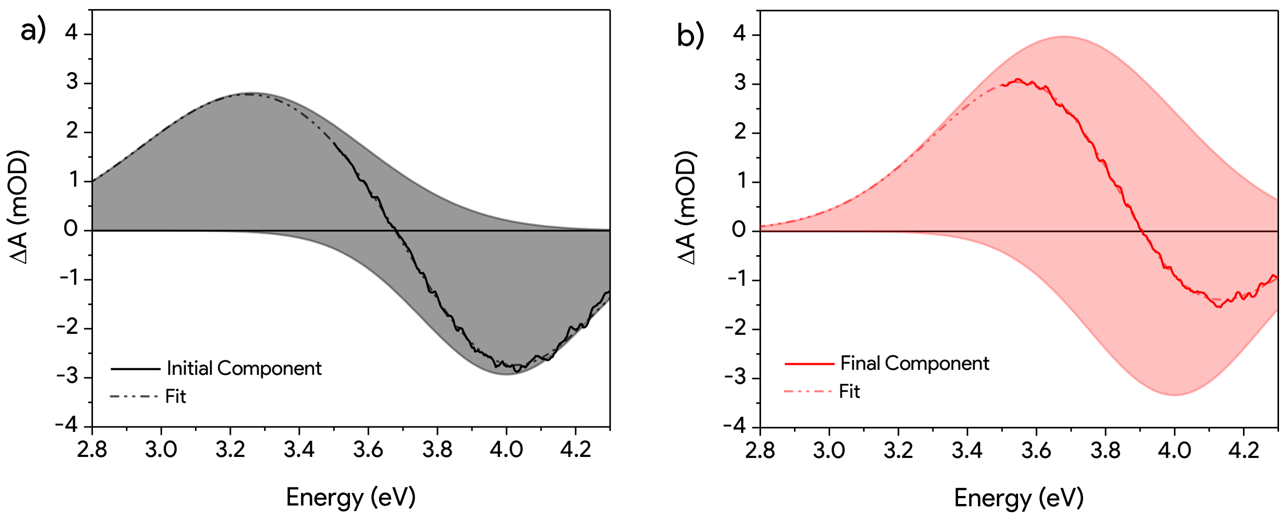

Figure S4 a. The black solid line is the initial component extracted using Glotaran while the dashed line represents the fit obtained using the two grey shaded gaussians. **b.** The red solid line is final spectral component extracted using Glotaran while the dashed line represents the fit obtained using the two shaded gaussians.

values we obtained from the final state spectral component (Figure S4b). Finally, the initial state component was fit as shown in Figure S4a. In table 1S the results of the fit are reported.

Analysis of the dynamics extracted with the Global Fit analysis

In order to evaluate the rise time and the decay time of the dynamics extracted using Glotaran, we use decay and a rise functions obtained by the convolution of a Gaussian peak (which simulates the IRF) with two exponentials. The final function is:

$$f(t) = A_0 * \frac{\tau_1}{\tau_2 - \tau_1} \cdot \left(e^{\frac{\sigma_{IRF}^2}{2\tau_2^2} - \frac{c+t}{\tau_2}} \cdot \left(1 + \mathrm{erf}\left(\frac{c+t}{\sqrt{2} \cdot \sigma_{IRF}} - \frac{\sigma_{IRF}}{\sqrt{2}\,\tau_2}\right)\right) - e^{\frac{\sigma_{IRF}^2}{2\tau_1^2} - \frac{c+t}{\tau_1}} \cdot \left(1 + \mathrm{erf}\left(\frac{c+t}{\sqrt{2} \cdot \sigma_{IRF}} - \frac{\sigma_{IRF}}{\sqrt{2}\,\tau_1}\right)\right)\right),$$

where τ_1 is the rise time of the signal, τ_2 the decay time of the signal and σ_{IRF} is the width of the pulse duration of the laser in a Gaussian approximation. The result of the fit is shown in Figure S5.

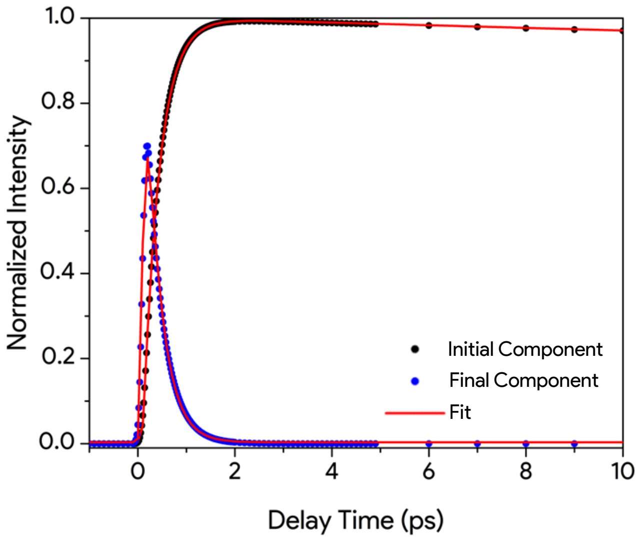

Figure S5 The dynamics of the components extracted from the global fit analysis and the fit obtained using the equation above.

REFERENCES

(1) Skála, T.; Šutara, F.; Prince, K. C.; Matolín, V. Cerium Oxide Stoichiometry Alteration via Sn Deposition: Influence of Temperature. *J. Electron Spectrosc. Relat. Phenom.* **2009**, *169* (1), 20–25. https://doi.org/10.1016/j.elspec.2008.10.003.

(2) Klingshirn, C.; Fallert, J.; Zhou, H.; Sartor, J.; Thiele, C.; Maier-Flaig, F.; Schneider, D.; Kalt, H. 65 Years of ZnO Research – Old and Very Recent Results. *Phys. Status Solidi B* **2010**, *247* (6), 1424–1447. https://doi.org/10.1002/pssb.200983195.

(3) Guo, S.; Arwin, H.; Jacobsen, S. N.; Järrendahl, K.; Helmersson, U. A Spectroscopic Ellipsometry Study of Cerium Dioxide Thin Films Grown on Sapphire by Rf Magnetron Sputtering. *J. Appl. Phys.* **1995**, *77* (10), 5369–5376. https://doi.org/10.1063/1.359225.

(4) Patsalas, P.; Logothetidis, S.; Sygellou, L.; Kennou, S. Structure-Dependent Electronic Properties of Nanocrystalline Cerium Oxide Films. *Phys. Rev. B - Condens. Matter Mater. Phys.* **2003**, *68* (3), 035104. https://doi.org/10.1103/PhysRevB.68.035104.

(5) Snellenburg, J. J.; Laptenok, S.; Seger, R.; Mullen, K. M.; Stokkum, I. H. M. van. Glotaran: A Java-Based Graphical User Interface for the R Package TIMP. *J. Stat. Softw.* **2012**, *49* (1), 1–22. https://doi.org/10.18637/jss.v049.i03.
